# Unveiling Equitable Surgical Prioritization: Insights From a Comprehensive Analysis Using the Medically Necessary and Time-Sensitive (MeNTS) Scoring System

**DOI:** 10.7759/cureus.74419

**Published:** 2024-11-25

**Authors:** Chrysanthy Ha, Nathan Carroll, Shawn Steen, Javier Romero, Graal Diaz

**Affiliations:** 1 Surgery, Community Memorial Hospital, Ventura, USA; 2 Family Medicine, Ventura County Medical Center, Ventura, USA; 3 Surgery, Ventura County Medical Center, Ventura, USA; 4 Medicine, Community Memorial Hospital, Ventura, USA

**Keywords:** clinical scoring system, covid-19, limited resources, racial and ethnic disparities, unequal access to healthcare

## Abstract

Background: This study addresses the intricate landscape of racial disparities in healthcare delivery, with a specific focus on surgical procedures. The concern was accentuated by the challenges posed during the COVID-19 pandemic when resources became scarce. Recognizing the potential impact of provider bias in medical decision-making, the American College of Surgeons introduced the Medically Necessary and Time-Sensitive (MeNTS) scoring system. This methodology aims to address procedures that, while not emergent, are deemed medically necessary and time-sensitive. This study analyzed whether using this scoring system decreased racial disparities between patients receiving surgery during the pandemic.

Methodology: A retrospective cross-sectional study was conducted using Electronic Medical Records from June 1, 2020, to December 31, 2021. We analyzed variations in MeNTS scores and time to surgery based on racial and ethnic backgrounds using bivariate and multivariate analyses.

Results: The analysis included 2,997 patients. Of these, 1,442 (42.84%) were Hispanic participants, 1,282 (38.09%) were non-Hispanic participants, and 642 (19.07%) were participants of other specified ethnic backgrounds. The racial composition comprised 2,955 (87.79%) White participants, 98 (2.91%) Asian participants, 50 (1.49%) African American participants, and 72 (2.14%) Alaska Native or American Indian participants. No significant differences in mean days to surgery or MeNTS scores were observed across racial and ethnic groups (Hispanic participants = 76.62 vs. non-Hispanic participants = 78.82, *P* = 0.8). A multivariate survival model showed that MeNTS scores below 30 were associated with higher surgery likelihood, with no significant disparities in race, ethnicity, or gender.

Conclusions: This comprehensive study utilizing the MeNTS scoring system reveals an absence of statistically significant racial disparities in surgical prioritization. These findings contribute valuable insights to the ongoing discourse surrounding equitable healthcare practices and emphasize the potential efficacy of standardized scoring systems in mitigating biases in medical decision-making.

## Introduction

Racial disparities in the U.S. healthcare system have been well documented. Since a landmark Institute of Medicine report in 2003 called on the nation to make an effort to reduce disparities in healthcare [1], multiple studies have contributed to our growing understanding of racial disparities. In 2011, the U.S. Department of Health and Human Services launched the largest federally based plan to improve healthcare equality for all races and ethnicities [2]. Recent studies have found that these disparities continue to persist [3-4].

Disparities also exist in surgical outcomes, with racial and ethnic minorities experiencing higher mortality and postoperative complications [5-8]. For instance, between 2012 and 2017, racial disparities in procedures like total knee arthroplasty persisted, with significantly fewer African American males undergoing surgery compared to White males [4]. This difference increased from a 105% to 124% difference. Shah et al. showed that after adjusting for covariates, including tumor stage, pancreatic tumor resection was less often recommended and performed in African American patients than in White patients (odds ratio [OR] = 0.88; confidence interval [CI], 0.82-0.95) [9]. Dong et al. reported that among patients with esophageal cancer matched for disease presentation, only 205 (10.8%) African American patients underwent surgery, compared with 433 (22.8%) White patients (*P* < 0.001) [10]. These statistics have not significantly changed in the past two decades.

The COVID-19 pandemic highlighted these healthcare disparities, with minorities experiencing higher infection and mortality rates. Early studies showed that African American individuals had a mortality rate twice as high as their proportion in the population in multiple states [11]. Hispanic or Latino individuals, Asian individuals, and Pacific Islander individuals experienced infection and death rates many times higher than those of White individuals but were only hospitalized at a slightly higher rate [12]. As resources became scarcer, including supplies of personal protective equipment and diversion of medical personnel to care for COVID-19-infected patients, access to medical care was severely limited and both inpatient and outpatient elective procedures were canceled. As the pandemic progressed, it was clear that healthcare institutions needed to resume elective surgical operations, but at a highly reduced rate. This period saw a disproportionate decrease in elective procedures for minority populations, exacerbating existing inequalities [13-14].

To address these disparities, the American College of Surgeons introduced the Medically Necessary and Time-Sensitive (MeNTS) scoring system. This system aims to prioritize surgical procedures based on medical necessity and urgency, minimizing provider bias [15]. The MeNTS score balances the case's urgency with the risk of COVID-19 transmission to hospital staff and measures objective data. We aim to determine if there are variations in MeNTS scores and time to surgery based on racial and ethnic backgrounds.

## Materials and methods

This was a retrospective cross-sectional study conducted at Ventura County Medical Center (VCMC), a 223-bed hospital with six operating rooms and 16 ICU beds. During the COVID pandemic from June 1, 2020, to December 31, 2021, VCMC adopted the MeNTS score as a requirement to schedule an elective surgery. It became a policy for the primary surgeon to calculate a MeNTS score for each patient at the time of the surgical consultation. The score consists of three domains: Procedure (Table 1), Disease (Table 2), and Patient (Table 3). The score for each domain was documented via discrete documentation elements in a *PowerForm* on the Oracle Cerner Millennium Electronic Medical Record, which would automatically calculate the total MeNTS score for each patient. The score total ranges from 21 to 105 points. Lower scores indicated higher priority.

**Table 1:** Medically Necessary Time-Sensitive (MeNTS) scoring worksheet: procedure factors. *Operating room (OR) time: physician time in OR; do not include setup/cleanup time. **Team size: count physicians, physician assistants; do not count other OR staff such as registered nurses, scrub technicians, anesthesiologists. ***OHNS: otolaryngology, head, and neck surgery

Procedure factors	1	2	3	4	5
OR time (minutes)*	<=30	31-60	61-120	121-180	≥181
Estimated length of stay	Outpatient	<23 hours	24-48 hours	≤ 3 days	>4 days
Postoperative intensive care unit need (%)	Very unlikely	<5%	5%-10%	11%-25%	≥25%
Estimated blood loss	<100 cc	101-250 cc	251-500 cc	501-750 cc	≥750 cc
Surgical team size**	1	2	3	4	>4
Intubation needed to perform procedure (probability), %	≤15	1%-5%	6%-10%	11%-25%	≥25%
Surgical site	None of the following	Abdominopelvic minimally invasive surgery	Abdominopelvic open surgery, infraumbilical	Abdominopelvic open surgery, supraumbilical	OHNS***/upper gastrointestinal/thoracic

**Table 2:** Medically Necessary Time-Sensitive (MeNTS) scoring worksheet: disease factors.

Disease factors	1	2	3	4	5
Non-operative treatment option effectiveness	Not available	Available, <40% as effective as surgery	Available, 40%-60% as effective as surgery	Available, 60%-95% as effective as surgery	Available, equally effective
Non-operative treatment option resource use/exposure risk	Significantly worse/not applicable	Somewhat worse	Equivalent	Somewhat better	Significantly better
Impact of two-week delay in disease outcome	Significantly worse	Worse	Moderately worse	Slightly worse	Minimally worse
Impact of two-week delay in surgical difficulty/risk	Significantly worse	Worse	Moderately worse	Slightly worse	Minimally worse
Impact of six-week delay in disease outcome	Significantly worse	Worse	Moderately worse	Slightly worse	Minimally worse
Impact of six-week delay in surgical difficulty/risk	Significantly worse	Worse	Moderately worse	Slightly worse	Minimally worse

**Table 3:** Medically Necessary Time-Sensitive (MeNTS) scoring worksheet: patient factors. *Includes asthma, chronic obstructive pulmonary disease, or cystic fibrosis. **Includes hypertension, congestive heart failure, or coronary artery disease. ***Includes hematologic malignancy, stem cell transplant, solid organ transplant, active/recent cytotoxic chemotherapy, anti-TNFα or other immunosuppressants, >20 mg prednisone equivalent/day, congenital immunodeficiency, hypogammaglobulinemia on Intravenous Immunoglobulin (IVIG), HIV with CD4 < 200. ****Includes fever, cough, sore throat, body aches, or diarrhea. CPAP, continuous positive airway pressure

Patient factors	1	2	3	4	5
Age (years)	<20	21-40	41-50	51-65	>65
Lung disease*	None	-	-	Minimal (rare inhaler)	>Minimal
Obstructive sleep apnea	Not present	-	-	Mild/moderate (no CPAP)	On CPAP
Cardiovascular disease**	None	Minimal (no meds)	Mild (≤ 1 med)	Moderate (2 meds)	Severe (≥3 meds)
Diabetes	None	-	Mild (no meds)	Moderate (PO meds only)	>Moderate (insulin)
Immunocompromised***	No			Moderate	Severe
Influenza-like illness symptoms****	None (Asymptomatic)	-	-	-	Yes
Exposure to a known COVID-19-positive person in past 14 days	No	Probably not	Possibly	Probably	Yes

The study included all adult patients (>18 years) who were scheduled for surgical procedures during the COVID-19 pandemic and had complete data. The main predictors of interest were ethnicity and race. Additional covariates included MeNTS scores, gender, and surgical specialty. The documentation elements and total scores along with relevant patient demographic and procedure data were extracted from the database via CCL query for analysis. Patients with incomplete data were excluded. Descriptive, bivariate, and multivariable analyses were performed using SAS 9.4 (SAS Institute Inc., Cary, NC). Survival analyses using Kaplan-Meier plots were conducted given its ability to manage censored data, handle time-to-event analysis, and compare different patient groups without relying on specific data distribution assumptions. Days-to-surgery was used as the final event, with statistical significance set at α < 0.05. The study was approved by the Ventura County Healthcare Agency Institutional Review Board. As the study used de-identified patient data, no additional consent was required. There was no funding involved in this study.

## Results

A total of 3,890 cases were scheduled during this time period, compared to 4,867 cases during a similar time period the year prior (20.1% reduction rate). A total of 2,997 patients had complete data and were included in this study (Table 4), with a mean age of 44.59 years (standard deviation [SD] 17.55). The racial composition was predominantly White (87.79%), with smaller percentages of Asian (2.91%), African American (1.49%), and Alaska Native or American Indian (2.14%) individuals. Of the patients, 42.84% identified as Hispanic, 38.09% as non-Hispanic, and 19.07% as individuals of other ethnic backgrounds. A total of 2,869 cases (85.23%) were elective, while 497 cases (14.77%) were urgent. The mean time to surgery was 76.72 days (SD = 127.93), and the mean total MeNTS score was 41.53 points (SD = 6.79) (Table 5). The surgical specialties consisted of general surgery (1,779, 58.81%), neurosurgery (37, 1.22%), obstetrics-gynecology (322, 10.64%), orthopedics (296, 9.79%), and other specialties (591, 19.54%). The general surgery patients had the highest average MeNTS score (41.58), while the neurosurgery patients had the lowest average MeNTS scores (37.54), and the longest average days to surgery (103.61).

**Table 4:** Study population summary.

Variable	Level	Count	Percentage
Race	White	2955	87.79%
	Other	191	5.67%
	AAPI	98	2.91%
	American Indian	72	2.14%
	Black or African American	50	1.49%
Ethnicity	Hispanic or Latino	1442	42.84%
	Non-Hispanic or Latino	1282	38.09%
	Other	642	19.07%
Sex	Female	2,046	60.78%
	Male	1,320	39.22%
Surgery completed	1	2,768	82.23%
	0	598	17.77%
Intubation probability	>=25%	2,162	64.23%
	<=1%	970	28.82%
	11%-25%	90	2.67%
	1%-5%	84	2.50%
	6%-10%	60	1.78%
Scheduled type	Elective	2,869	85.23%
	Urgent	497	14.77%

**Table 5:** Days to surgery and total MeNTS score by specialty. MeNTS, Medically Necessary and Time-Sensitive

Specialty	Count	Percentage	Days to surgery, mean (SD)	Total MeNTS score, mean (SD)
General surgery	1,779	58.81%	73.87 (14.16)	41.58 (19.34)
Neurosurgery	37	1.22%	103.61 (32.07)	37.54 (17.12)
Obstetrics-gynecology	322	10.64%	92.56 (27.17)	43.36 (21.41)
Orthopedics	296	9.79%	93.08 (31.16)	40.29 (19.01)
Other	591	19.54%	64.79 (11.48)	41.13 (31.32)

**Figure 1:**
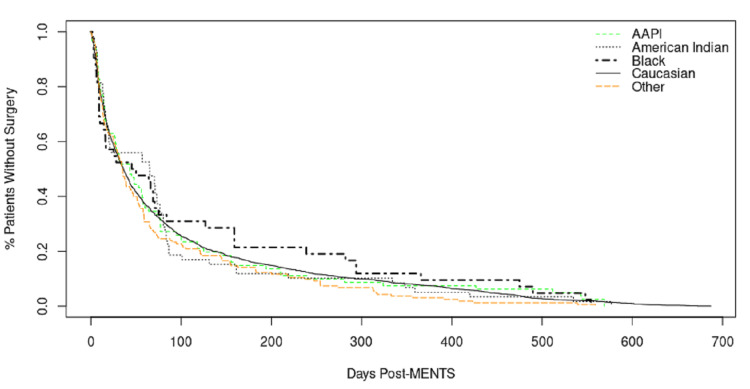
Mean days to surgery by race. AAPI, Asian American Pacific Islander; MeNTS, Medically Necessary and Time-Sensitive

Bivariate linear regression, examining MeNTS scores in relation to race, sex, ethnicity, and intubation status, demonstrated no statistically significant differences in mean days to surgery by race (Table 6) and ethnicity (Hispanic participants = 76.62 vs. non-Hispanic participants = 78.82, *P* = 0.8) (Table 7). A multivariate survival model demonstrated similar results with no statistical difference in mean days to surgery by race (Figure 1). MeNTS scores below 30 were associated with a higher likelihood of undergoing surgery, with no significant differences by race, ethnicity, or gender.

**Table 6:** Survival analysis of time to surgery by race.

Race	HR	T	*P*-value	Confidence interval
American Indian	0.99	-0.03	0.98	0.4-2.46
Black or African American	1.85	1.44	0.15	0.8-4.26
Other	0.84	-0.44	0.66	0.4-1.8
White	1.42	1.15	0.25	0.78-2.58

**Table 7:** Survival analysis of time to surgery by ethnicity. HR, hazard ratio

Ethnicity	HR	T	*P*-value	Confidence interval
Non-Hispanic or Latino	1.02	0.25	0.8	0.85-1.23
Hispanic or Latino	0.65	-3.2	0	0.49-0.84

## Discussion

During the first 18 months of the COVID pandemic, there were nearly 1,000 fewer surgeries scheduled as compared to a similar timeframe in the pre-COVID era. Despite the decrease in scheduled surgeries, this study demonstrated no statistically significant racial disparities in surgical prioritization when using the MeNTS scoring system. These findings suggest that standardized scoring can help mitigate provider bias in medical decision-making. In addition to using an objective scoring system to decrease racial disparities, the study also shows the utility of a scoring system to ensure equal access to care when resources are scarce.

Racial disparities in healthcare have been well-documented, with minorities often experiencing worse outcomes. As one of the four principles of medical ethics, the principle of justice calls for physicians to take social accountability and develop strategies to reduce those disparities. The situation is further complicated when resources are scarce, and denying a patient a needed surgery can be viewed as a violation of the principle of beneficence. Public health is often a balance between the principles of social justice and beneficence. One of the strengths of the MeNTS scoring system is that it addresses both these principles. The Disease score takes into account the impact on the patient if the needed surgery is delayed. The Procedure and Patient scores address the impact on the system and best use of resources, particularly if a provider were to become infected with COVID-19, making a scarce resource even scarcer. This study adds to the evidence by showing that the MeNTS system can promote equitable surgical prioritization during resource-constrained situations like the COVID-19 pandemic.

Within the medical care system, there are multiple avenues along which racial disparities can manifest, including pre-operative, perioperative, and postoperative encounters [16]. These avenues are also ways providers can intervene and implement purposeful changes to address racial disparities. Along the pre-operative route, racial minorities often have unequal access to care and screening. Disparities in care processes, such as referrals, are linked to racial status, and increasing access improves surgical outcomes by addressing health issues more promptly [17]. The disparity in referrals and the subsequent decision to offer surgery can be attributed in part to the unconscious bias of providers. It is well-studied that implicit bias in physicians may cause miscommunication in the severity of symptoms reported by minority patients and create dependence on stereotypes to make clinical decisions in a time-constrained environment [18-19]. Training physicians in implicit bias has been shown to enhance cultural competence [20]. The implementation of the MeNTS scoring system in various healthcare settings could help ensure fair resource allocation and reduce disparities. Healthcare institutions should consider adopting this system to enhance equity in surgical care.

This study has several notable strengths, including a large and diverse sample size that enhances the generalizability of the findings. The use of the standardized MeNTS scoring system is a significant strength, as it helps to reduce provider bias in surgical prioritization. Additionally, the comprehensive statistical analyses, including survival analysis and the use of a multivariate model, provide robust insights into the factors influencing surgical wait times and prioritization. However, some limitations must be considered. The study's single-site design may limit the generalizability of the results to other settings or populations. The exclusion of 379 patients with incomplete data (11% of the sample) could introduce bias, potentially affecting the study's findings. Moreover, while the study adjusted for several key confounders, there may be other unmeasured variables that could influence the results. Future research should aim to validate these findings in different settings and with larger, more diverse populations to further assess the effectiveness of the MeNTS scoring system in reducing racial disparities in surgical care.

## Conclusions

In conclusion, the MeNTS scoring system shows promise in promoting equitable surgical prioritization and mitigating racial disparities. Its broader implementation could significantly improve fairness in healthcare delivery. While this scoring system is specific to COVID-19 and respiratory infections in general, it can be modified to be used in numerous other situations when the surgical need is significantly higher than resource availability, such as during humanitarian crises or in the early phases of natural disasters. If implemented early, it can help determine fair resource allocation and ensure maximum systems efficiency. Further studies are needed to validate the MeNTS system in different populations and healthcare settings. This will help to confirm the system's utility in promoting equitable healthcare practices across different contexts. Research should also explore its application in various surgical specialties and its impact on long-term outcomes.
